# Improved cortical activity and reduced gait asymmetry during poststroke self-paced walking rehabilitation

**DOI:** 10.1186/s12984-021-00859-7

**Published:** 2021-04-13

**Authors:** Keonyoung Oh, Jihong Park, Seong Hyeon Jo, Seong-Jin Hong, Won-Seok Kim, Nam-Jong Paik, Hyung-Soon Park

**Affiliations:** 1 Arms & Hands Lab, Shirley Ryan AbilityLab, Chicago, IL USA; 2 Department of Mechanical Engineering, Korea Advanced Institute of Science and Technology (KAIST), 291 Daehak-ro, Yuseong-gu, Daejeon, 34141 Republic of Korea; 3 Department of Rehabilitation, Seoul National University Bundang Hospital, 82, Gumi-ro 173 Beon-gil, Bundang-gu, Seongnam-si, Gyeonggi-do 13620 Republic of Korea

**Keywords:** Gait training, Self-paced treadmill, Stroke rehabilitation, Electroencephalography, Gait asymmetry, Cortical activation

## Abstract

**Background:**

For patients with gait impairment due to neurological disorders, body weight-supported treadmill training (BWSTT) has been widely used for gait rehabilitation. On a conventional (passive) treadmill that runs at a constant speed, however, the level of patient engagement and cortical activity decreased compared with gait training on the ground. To increase the level of cognitive engagement and brain activity during gait rehabilitation, a self-paced (active) treadmill is introduced to allow patients to actively control walking speed, as with overground walking.

**Methods:**

To validate the effects of self-paced treadmill walking on cortical activities, this paper presents a clinical test with stroke survivors. We hypothesized that cortical activities on the affected side of the brain would also increase during active walking because patients have to match the target walking speed with the affected lower limbs. Thus, asymmetric gait patterns such as limping or hobbling might also decrease during active walking.

**Results:**

Although the clinical test was conducted in a short period, the patients showed higher cognitive engagement, improved brain activities assessed by electroencephalography (EEG), and decreased gait asymmetry with the self-paced treadmill. As expected, increases in the spectral power of the low γ and β bands in the prefrontal cortex (PFC), premotor cortex (PMC), and supramarginal gyrus (SG) were found, which are possibly related to processing sensory data and planning voluntary movements. In addition, these changes in cortical activities were also found with the affected lower limbs during the swing phase. Since our treadmill controller tracked the swing speed of the leg to control walking speed, such results imply that subjects made substantial effort to control their affected legs in the swing phase to match the target walking speed.

**Conclusions:**

The patients also showed reduced gait asymmetry patterns. Based on the results, the self-paced gait training system has the potential to train the symmetric gait and to promote the related cortical activities after stroke.

*Trial registration* Not applicable

## Background

Stroke is a prevalent disease caused by hemorrhagic or ischemic injury in the brain and accompanied by motor disability. Impaired motor function can be recovered to some extent by cortical reorganization [1]. Rehabilitation within the first 3 months after stroke is essential to promote recovery by neural plasticity [2]. The restoration of locomotor function is a major issue in rehabilitation for many patients affected by stroke since gait disorders limit performing social and daily living activities [3]. In addition, the risk of falls that could cause traumatic injury increases as gait performance decreases [4]. Thus, in the hospital and afterwards, gait rehabilitation is provided for stroke survivors to improve their walking performance [5, 6].

Body weight-supported treadmill training (BWSTT) is widely performed for gait rehabilitation since it provides safe and repeatable training in small spaces with less burden on the therapist compared with overground training. However, there is a study showing that the effect of training on a treadmill was not superior to that of overground training with a therapist [7]. On the conventional treadmill, patients have difficulties voluntarily participating and engaging in training because they adapt to the unchanging speed. To overcome this limitation of the traditional treadmill, a self-paced treadmill that simulates overground walking was developed [8–11], and sometimes with a virtual reality system [12, 13] for gait rehabilitation after neurological disorders [10, 12, 13]. The speed of the treadmill belt is controlled by preferences of a user by measuring the position of the user’s body segments [11, 14] using a commercial depth sensor [15] or using a motion capture system [16]. In addition, unexpected inertial force by sudden acceleration and deceleration was modified by the velocity of the swing foot [9].

Several studies reported differences in a variability of spatiotemporal gait parameters between the self-paced treadmill walking and the conventional treadmill walking with a fixed speed. A larger variability of stride length and stride time was found with the self-paced treadmill than the conventional treadmill in neurologically intact human subjects [17, 18]. When a control algorithm of the self-paced treadmill is adjusted to be more sensitive to a user’s movement, larger variabilities of these gait parameters are found [19]. In addition, such spatiotemporal gait parameters on self-paced treadmill can be effectively used for Multiple Sclerosis prediction [20]. As far as the authors know, however, it remains unclear how the self-paced treadmill walking affects cortical activities in patients after neurological disorders.

Monitoring the brain activity of the patient is helpful in identifying the effect of training and suggests an effective gait rehabilitation approach since impaired function could be recovered by cortical reorganization [21]. Several studies have shown that the function and structure in the regions adjacent to the impaired area after stroke, as well as regions remote from the cortical lesion, were changed during the recovery process by brain plasticity [22]. In addition to the voluntary recovery of lost function during the initial several months after stroke, therapy-induced neural reorganization could promote recovery [23, 24].

There are several noninvasive neuroimaging techniques to monitor cortical activity, such as fMRI, fNIRS, and EEG. Functional magnetic resonance imaging (fMRI) is widely used to elucidate brain activation due to its high spatial resolution. Although the activity in the brain, including deep brain tissue and the cerebral cortex, can be monitored by fMRI, this neuroimaging technology is not suitable to study cortical activity during dynamic walking since the subject has to remain in the lying position in a static state before or after the intervention [25]. To monitor cortical activity during dynamic activities, functional near-infrared spectroscopy (fNIRS) and electroencephalography (EEG) are suitable to examine cortical changes during walking. Increased oxygenated hemoglobin (oxyHb) in sensorimotor cortices and supplementary cortex was monitored using fNIRS during walking [26, 27]. In another study, activation in the prefrontal, premotor and sensorimotor cortex was reported to increase while walking [28]. During precise stepping, increased oxyHb in the prefrontal and supplementary motor cortex was observed in another study [29]. In research with participants performing a dual task during walking, a change in activity in the prefrontal cortex related to cognitive performance was observed [30].

Although EEG recording has a low spatial resolution, intra-stride characteristics could be identified due to its high temporal resolution. Previous studies have reported that spectral power decreased in the $\upmu$ and $\upbeta$ bands in the sensorimotor cortex are observed during movement [31, 32]. These characteristics are referred to as event-related desynchronization (ERD). One study elucidated that the intra-stride patterns of cortical activity in the anterior cingulate, posterior parietal, and sensorimotor cortex were coupled to the gait phase during steady walking on a treadmill [28]. In another study, significant ERD in the $\upbeta$ band and low $\upgamma$ band was demonstrated during active walking [33]. EEG can also be used to demonstrate effective connectivity by calculating Granger causality [34, 35]. In one study, connectivity between motor areas and others decreased, but connectivity between nonmotor areas increased during steady walking compared to standing [36].

In this study, we used a similar experimental protocol as that used with healthy subjects in the previous study to extend the analysis to stroke survivors [33]. Brain activity levels were monitored and compared between the self-paced treadmill and conventional treadmill with the fixed walking speed using EEG recording. We hypothesized that active walking on the self-paced treadmill allows patients to engage more in the training and to increase cortical activity than walking at a fixed speed. In addition, gait asymmetry patterns might decrease during active walking, as target walking speed is actively maintained, compared to passive walking conditions.

## Methods

Participants with postischemic stroke hemiplegia who could provide consent and adhere to the study protocol without any apparent cognitive impairment were prospectively screened for the ability to walk without manual assistance and recruited from the Seoul National University Bundang Hospital (SNUBH), a tertiary teaching hospital located in Seongnam, Korea, from December 2016 to September 2017. They were excluded if they scored below three on the Functional Ambulation Categories (FAC) [37, 38]. The institutional review board of SNUBH approved the study protocol (IRB no. B-1608/358-004), and written informed consent was obtained from all the participants.

**Fig. 1:**
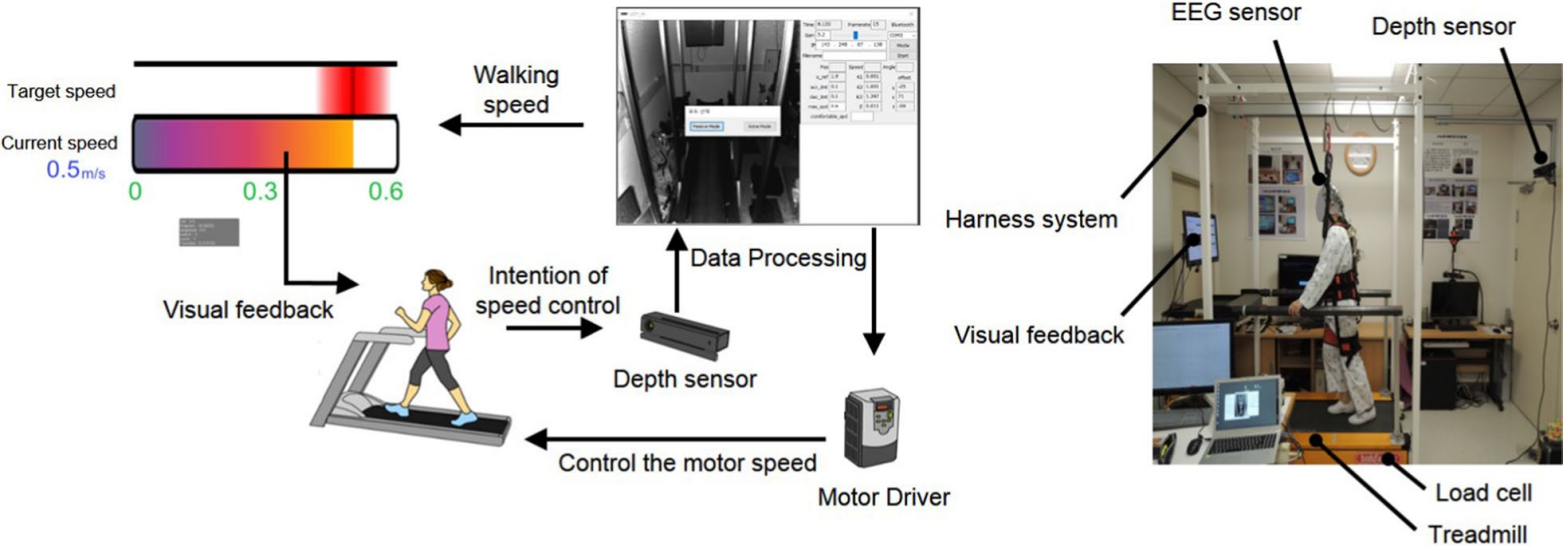
Control of the self-paced (active) treadmill (left) and experimental setup (right)

The self-paced treadmill was controlled by recognizing a user’s intention through acceleration or deceleration (Fig. 1) [9, 15]. The positions and velocities of the user’s pelvis and foot were measured using KINECT (Microsoft, Redmond, WA, US), and the data were processed by MFC (Microsoft, Redmond, WA, US). If a user wanted to walk faster and moved forward with a faster swing leg, the controller recognized this intention and accelerated the belt. For active walking, both the target speed and the current speed controlled by the user’s intention of speed were displayed on the monitor, so the user could change their speed to adjust to the target speed.

All participants were evaluated with the FAC first, followed by the Berg balance scale (BBS) [39], Falls Efficacy Scale (FES) [40] and 10-m walking test (10 mWT), and relevant demographic data, such as age and medical history, were obtained through an interview conducted at the beginning of the session.

The participants performed two active and two passive walking sessions in random order. Each walking session consisted of a set of slow walking and fast walking trials, which were repeated nine times. Between each session, a minimum of a 5-min break was provided to avoid fatigue effects. EEG data were collected while participants performed the walking trials. For the safety of the subjects, the fast and slow speeds in the walking sessions were determined not to exceed the maximum walking speed measured from the 10-m walking test and were set to 70% and 40% of the maximum walking speed from the 10-m walking test.

To identify walking phases, four one-dimensional load cells (DSCK-500, Bongshin, Osan, South Korea) were implemented in each corner of the treadmill. From measured vertical ground reaction forces (GRF), instants of heel-strikes (HS) and toe-off (TO) were calculated when the GRF exceeded and dropped below a threshold of 20N. To investigate the gait asymmetry patterns, gait phase durations were also calculated from such gait indices. The GRF data were collected at 1080 Hz and then filtered with a low-pass filter with a cutoff frequency of 20 Hz. Gait asymmetry was estimated by calculating the difference in stance duration between the two lower limb sides and then compared between the active and passive walking using the paired t-test. Since the walking speed was fixed for the passive walking session with zero variance, the walking speeds between the active and passive walking conditions were compared using the Wilcoxon Rank Sum test.

### Collecting EEG data and signal processing

To assess the level of activity in each cortical region, EEG data were collected using actiCHamp (Brain Vision, Morrisville, NC, United States). A total of 64 channels were located on a subject’s scalp, and the data were collected at 1080 Hz. Postprocessing of EEG data using EEGlab (Swartz Center for Computational Neuroscience, Lo Jolla, CA, United States) based on MATLAB (Mathworks, Natick, MA, United States) toolbox was conducted. Details on the postprocessing procedures were adapted from and can be found in the previous research [33].

First, all the signals from the walking trials and the rest periods were combined in a single data set. A Butterworth 5th order high-pass filter was then applied with a cutoff frequency of 1 Hz. To eliminate noisy data, signals containing a standard deviation (SD) larger than 1000 μV or kurtosis larger than five SD were removed [28]. After removing noisy data, the EEG signals were rereferenced to have a common average value. To remove a signal contaminated with artifacts, an artifact subspace reconstruction (ASR) function [41] from the EEGlab was conducted. Compared to the relatively clean signals during the 2 min of rest without any kinematic change, a statistical test was conducted with a moving window (500 ms) of a single channel to find signals that have variance larger than a threshold of three SD, and such signals were identified as corrupted data.

Since any single channel could be affected by neighboring channels and nonbrain signals, an extended independent component analysis (EICA) [42–44] was conducted to determine the independent components (IC) (or signals) from only brain sources. After the Adaptive mixture independent component analysis (AMICA), equivalent current dipole sources were found using the DIPFIT function in EEGlab [45]. Dipoles accounting for less than 20% of the variance of the calculated IC projection on the scalp were neglected, and other dipoles were separated into nonbrain and brain sources according to their locations and power spectra. The clustering of ICs was then conducted for all the subjects using the power spectral density and the location of the dipole. A k-means clustering method was used to make the clusters, and ICs having variance larger than three SDs were identified as outliers and neglected for the analysis. The Brodmann areas of the estimated clusters were identified using the Talairach atlas [46].

To perform a temporal analysis of the EEG data, the EEG data were synchronized from the calculated gait phase indices. From all the points that were in the 0.1-s period before the right heel strike (RHS), epochs were obtained to have a minimum of one gait stride from the RHS to the following RHS. Such epochs were then categorized into four conditions: slow, fast, accelerated, and decelerated walking for both active and passive walking tasks. Within each epoch, a time-warping function was performed for all indices (RHS, LTO, LHS, RTO, and RHS) that have the same latencies from the start of the stride.

To investigate intra-stride cortical activity, the power spectral density with respect to each gait index, called event-related spectral perturbations (ERSP), was calculated. All the ERSPs from the ICs in a cluster were calculated first and then averaged to obtain the grand mean ERSP in a cluster. Each frequency component of such time–frequency decomposition was then normalized to an average value of the frequency component itself. A significance test and masking in the ERSP were conducted using a bootstrap function in the EEGlab.

To compare the cortical activities between active and passive walking, event-related desynchronization (ERD) and synchronization (ERS) were computed. Before comparing ERD/ERS in each cluster from the two conditions, the result from the time–frequency decomposition with the rest period dataset in the same cluster was subtracted to act as a baseline. A direct comparison of the level of activation was then conducted by calculating a difference in the ERD/ERS from two conditions. Statistical differences in ERSPs between the active and passive walking sessions were determined using the non-parametric bootstrapping method implemented in the EEGlab [43], with a significance level of 0.05.

## Results

**Fig. 2:**
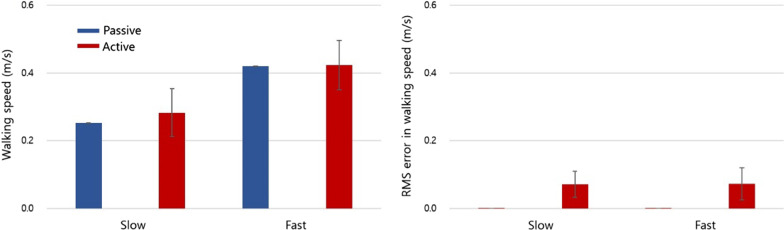
Walking speeds (left) and RMS error in speeds (right) referenced to the target speed for all subjects

Seven (four males and three females; 67.85 ± 9.40 years old) stroke survivors participated this study and were enlisted for the analysis (Table 2). The average slow and fast walking speeds during the active walking sessions were 0.28 ± 0.14 m/s and 0.42 ± 0.15 m/s, respectively, and 0.25 ± 0.00 m/s and 0.42 ± 0.00 m/s during the passive walking session (Fig. 2). There was no significant difference in the walking speeds between the passive and active walking sessions.

**Fig. 3:**
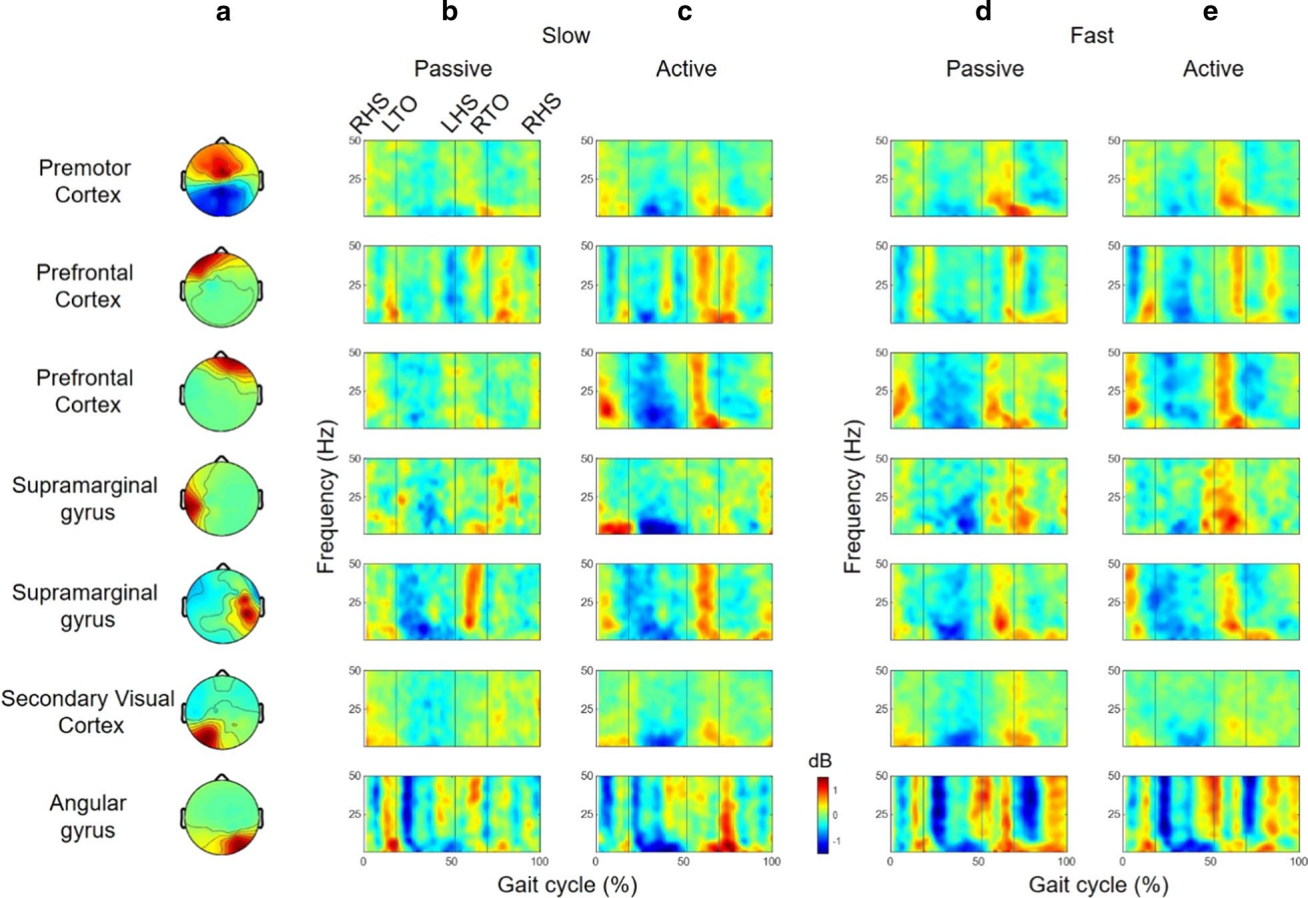
**a** The topographical maps of the estimated clusters and the ERSP from each cluster for **b** the passive and **c** active walking trials at the slow speed from all subjects. The ERSP for **d** the passive and active walking trials at the fast speed. The vertical lines in the ERSP plots indicate gait phase indices (RHO, LTO, LHS, and RTO)

The k-means clustering resulted in seven clusters (Fig. 3a and Table 1). The Brodmann area, numbers of ICs, and subjects in each cluster are represented in Table 1. ERSPs from all the clusters computed showed periodic fluctuations as the gait cycle passed (Fig. 3). The spectral power of the β and γ frequency bands from the PMC, PFC, and SG increased in the double support and early swing phase (Fig. 3).

**Table 1:**
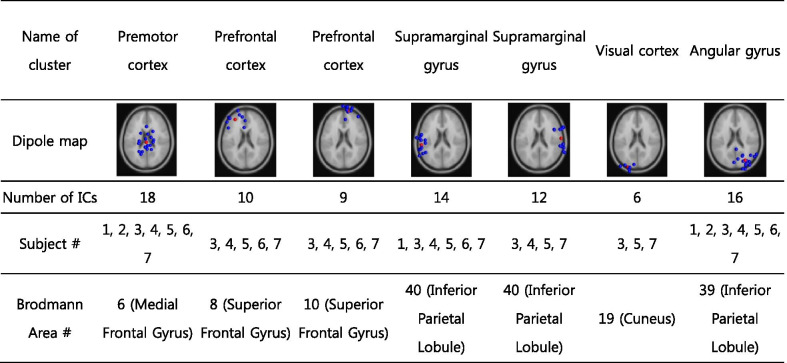
Calculated dipoles (electrical sources) and clusters

**Fig. 4:**
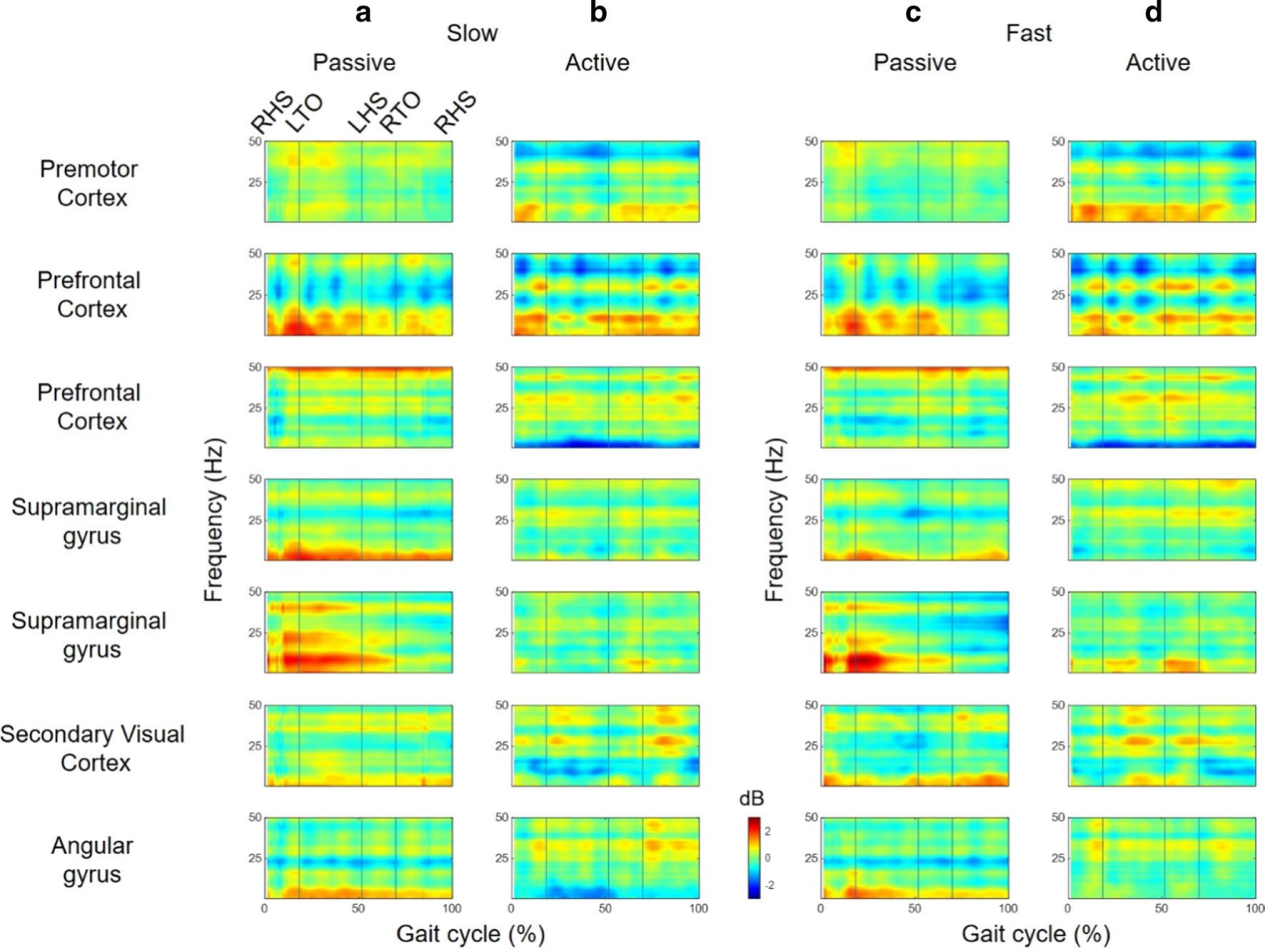
The ERD/ERS (active–passive) from the estimated clusters during a stride from all subjects for **a** the passive and **b** active walking trials at the slow walking speed and **c** passive and **d** active walking trials at the fast walking speed. The vertical lines in the ERSP plots indicate gait phase indices (RHO, LTO, LHS, and RTO)

**Fig. 5:**
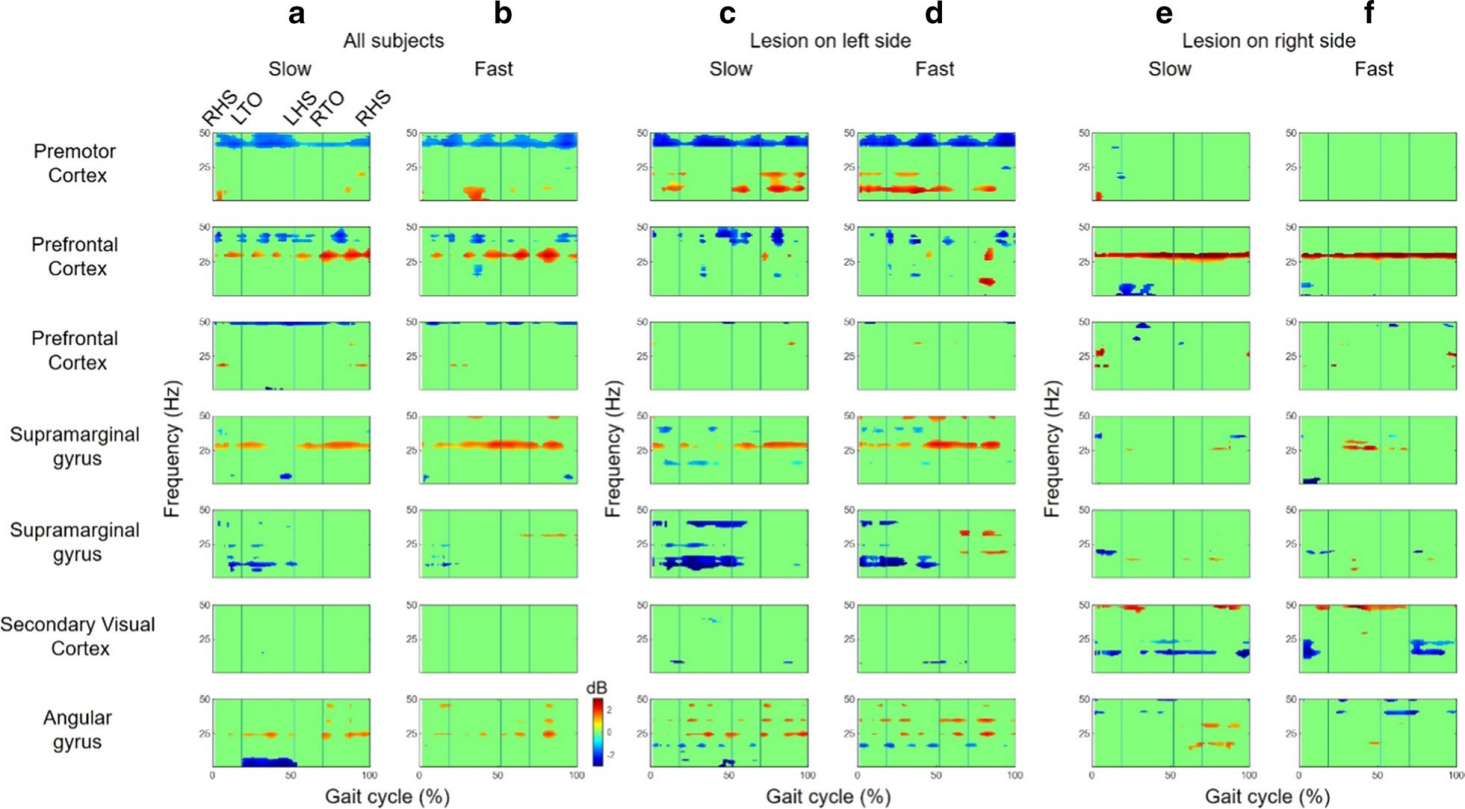
Differences in ERD/ERS (active–passive) from the estimated clusters during a stride from **a**, **b** all subjects and subjects with brain lesions on **c**, **d** the left and **e**, **f** right side of their brain. For each group, the left and right columns indicate the slow and fast walking trials, respectively. The vertical lines in the ERSP plots indicate gait phase indices (RHO, LTO, LHS, and RTO). Statistically nonsignificant differences (p > 0.05) were masked with zero, as shown in light green

Compared to the passive walking, the active walking aroused enhanced synchronization and desynchronization in some cortices. The lower γ (30–50 Hz) band desynchronizations in the PMC were significantly enhanced in the active walking trials (Figs. 4 and 5). The upper β (14–30 Hz) band synchronizations were enhanced in the PFC and SG (Figs. 4 and 5). The μ band desynchronizations were enhanced in the SG and the visual cortex (Figs. 4 and 5).

Such changes in cortical activities were significant, even in the affected side of brain. The subjects with the lesion on the left side of their brain showed enhanced upper β band synchronizations in the PFC and SG at both walking speeds (Fig. 5c, d and Table 2). The subjects with the lesion on the right side of their brain also showed significantly enhanced β band synchronizations in the PFC (Fig. 5e, f and Table 2).

**Table 2:** Demographics and clinical scores for the poststroke hemiplegic patients (*ACA* anterior cerebral artery, *MCA* middle cerebral artery, *PCA* posterior cerebral artery)

Subject number	Sex	Age	Location of infarcts	Time since stroke onset	FAC	BBS	FES	10 mWT
1	Female	60	Left MCA territory	13 days	5	56	65	11.7
2	Male	66	Multiple lacunes	2.5 years	4	52	89	24.0
3	Male	84	Left MCA and ACA territory	21 days	4	46	106	11.87
4	Female	75	Right MCA territory	26 years	5	56	82	15.67
5	Male	73	Right MCA territory	13 days	3	49	60	11.87
6	Female	54	Left PCA territory	11 days	5	55	4	8.08
7	Male	63	Left MCA territory	9 days	3	55	13	6.74

**Fig. 6:**
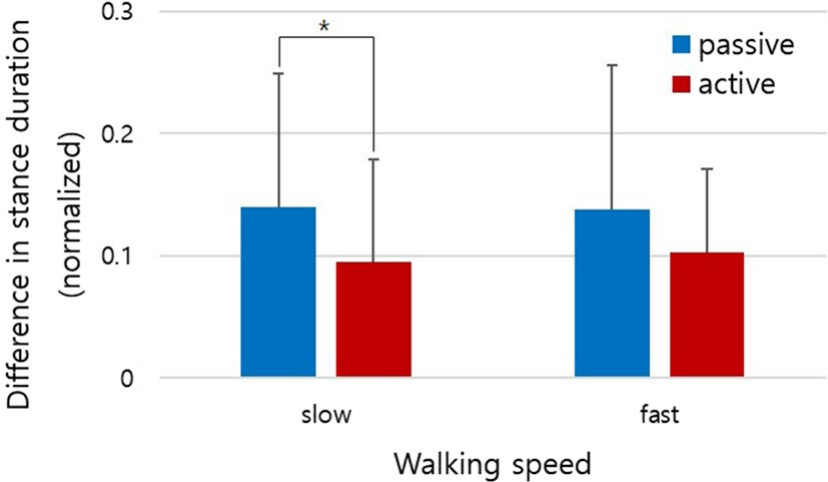
Gait asymmetries (differences in stance duration) between the passive and active walking tasks from all subjects. All values were normalized to each respective mean stance duration. (*p < 0.05)

Gait asymmetries due to hemiparesis also decreased during the active treadmill trials. The participants showed decreased asymmetry by an average of 32.0% and 25.7% during slow (p = 0.032) and fast (p = 0.057) walking speeds, respectively (Fig. 6).

## Discussion

The stroke survivors on the self-paced treadmill described above showed increased cortical activities in their brains compared to the conventional passive treadmill. Such cortical changes were significant in the visual cortex, premotor cortex, supplementary motor cortex, and somatosensory cortex, especially when the affected lower limb was in the late-swing phase and immediately before its heel strike. Since the controller implemented in our active treadmill system tracked the speed of the swing leg to estimate the user’s gait speed and to set the target speed, the increases in cortical activation level implied that subjects paid substantial attention to the target speed and tried to adjust the speed of their affected legs in the swing phase to match the target walking speed given by the self-paced treadmill system. Possible explanations of the increased levels of cortical activation in each region of cortex is further discussed below.

### Activation in the prefrontal cortex

The prefrontal cortex showed a higher activation level during active walking, and it could be presumed that subjects paid more attention to processing visual feedback on the velocity error and trying to maintain the given target speed as instructed. The processing of visual feedback and the corresponding greater control of their body joints might have imposed an additional task for the participants. Based on previous studies that reported that the prefrontal cortex might be relevant to gaze orientation, error detection, sensory reweighting and feedback, and executive functions, the subjects seemed to try to pay attention to performing such tasks. During human balance control with external perturbation, greater activation in the PFC was found and interpreted as an effort to assess the error through visuospatial attention [47]. To detect error between the target and the subject’s speed, similar cortical involvement processing visuospatial information might be engaged. All the subjects who participated in this experiment understood the task and performed well, so it is possible to speculate that the subjects did not have severe injury in the PFC. Because the PFC may also contribute to orchestrating internal goals and means [48], if the PFC is impaired after stroke, the subjects might find it difficult to understand and repeatedly perform the two different tasks. Moreover, the PFC is also known to carry out cross-modal sensory processing, and such involvement could be seen if we look at the EEG signal within the gamma band. During active walking, such a gamma band showed a higher activation level, so it may be relevant to combining visual, spatial, and possibly other sensory information.

### Activation in the supplementary motor area

Following cortical involvement in the PFC, which assesses visuospatial information, the involvement and higher activation levels in the supplementary motor cortex might be relevant to conducting tasks by programming sequential and complex locomotor mechanisms. Recently, a number of studies have been conducted to reveal the role of the SMC, and these studies have suggested that the SMC could provide a bridge between the intention in mind and voluntary external movement [49] and could organize motor behavior [50]. Significant activation in the SMC was found before movement in the fingers of human subjects, and the activation preceded cortical involvement in the PMA, which is known to control actual movements [51]. Rather than the posterior part of the SMC, it was the anterior part that was relevant to preparing for the motion. Once the subject was asked to follow the target speed, they had to assess the error between their actual speed and the target speed and decide whether they needed to accelerate or decelerate. Differing from steady-state (or fixed-speed) walking, the subjects might be asked to make decisions regarding such transient gait speed and prepare subsequent movement, including faster/slower swing of the leg or larger/smaller magnitude of propulsion forces. Such preparation for sequential movements may be related to greater activation in the SMC. Some researchers have suggested that the SMC also played a role in preparing and performing complex movements, such as gait initiation [52], which could be supported by our results that the SMC was highly activated when the subjects were asked to prepare and perform a relatively complex transient gait rather than steady walking.

### Cortical involvement of the somatosensory cortex

The patients engaged greater cortical effort to process sensory information from their limbs during active walking than during passive walking. Although both speeds were not significantly different during passive and active walking tasks (Fig. 2), the belt speed was controlled by tracking the pelvis position and the speed of the swing leg, so the subjects needed to control their lower limbs precisely to match the given target speed. Oscillatory power in the beta band was also increased in the somatosensory cortex in the supramarginal gyrus, which implied that there was an increase in neural populations recruited during active walking. To date, several studies have reported that the somatosensory cortex in the supramarginal gyrus contributes to body positioning, especially upright positioning [53], finger positioning [54], and body displacement perceptions [55]. In addition, the β band (13–30 Hz) in the sensorimotor cortex has been shown to become activated when movements occur [31, 56, 57]. Moreover, such β band power and connectivity between the sensorimotor cortex and the parietal cortex were shown to have larger sensory feedback gain, as it was very important to perform accurate movement [58]. As movement selection demands vary, the cortex may distribute the activity of the neural population [59, 60] in alpha and beta bands. To distinguish the role of alpha and beta band activities in the sensorimotor cortex from each other, a virtual task that involved imagining grasping a bar was conducted, and a larger population in the beta band was directly connected to the higher movement selection demand [59, 60]. Based on such studies, higher activity in the beta band from the sensorimotor cortex during active walking may imply that the suggested active walking system demands a larger population to decide and conduct voluntary movements.

### Gait asymmetry decreased

The increased level of cortical activity levels might be mostly relevant to the swing movement of the affected legs and the simultaneous balance control during the single support phase. Since the participants were asked to match the target walking speed even with the affected lower limb in the stance phase, they needed to lengthen the stance duration with the affected leg, and they were somewhat forced to lengthen the swing movements. In line with this speculation, a recent study reported that self-paced treadmill walking resulted in a larger intra-stride walking speed variation and a correspondingly higher risk of falls [61], which probably required additional effort to control balance with the affected side of the lower limb. When the affected leg was in stance during passive walking, subjects showed a 28.8% decrease in stance duration during the active walking session. In addition, previous studies have revealed that most energy loss and compensation occurred especially during the double support phase [62, 63], so sophisticated body control and corresponding high levels of cortical activities were required [28] to maintain body balance and walking speed in the double support phase. If some cortical regions, however, did not have intact control of the affected leg, the healthy leg would share the burden of balancing and propulsion, and gait asymmetry would occur. Higher activation levels and phasic synchronization in the PFC and SG on the affected side of brain during active walking would result in better control when standing, and propulsion would occur. Interestingly, as predicted, the affected leg contacted the ground earlier and propelled the body for a longer duration. As a result, gait asymmetry, defined as a difference in stance duration between the healthy and the affected leg, decreased by approximately 28.8% from all the subjects during the active walking trials (Fig. 6) compared to the passive walking trials. In addition to the higher cortical activities and phasic correlation found in this study, connectivity between such cortices has to be identified and assessed to ensure that such phasic synchronization and high activation result in improved networking between the cortices and the subsequently reduced gait asymmetry.

Notably, subjects with lesions on the right side of their brain showed significant increases in cortical activation levels but relatively less than the group with the lesion on the opposite side of the brain. First, a source of this inconsistency might have come from the limited number of subjects. In addition, compared to stroke survivors with lesions in their left brain (13.5 ± 5.26 days of stroke onset), the right-side lesion group had a significantly longer onset period (9.18 ± 13.76 years of stroke onset). It is worth noting that one of the participants (subject #4 from Table 2) has a significantly longer onset time of stroke of 26 years than other participants, so it may limit a possibility to determine possible effects of timing of gait training after stroke on cortical activities. Earlier timing of the gait rehabilitation might impact cortical activities [64]; however, our study was not able to determine the effects of the rehabilitation timing on the corresponding neuroplasticity due to the limited number of subjects and inconsistent onset time of stroke. and this possibility needs to be further investigated.

### CPG

Although the cortices and their connectivity need to be highly activated to promote neuroplasticity in patients with brain lesions, some previous studies have pointed out that walking at a constant speed, such as passive walking, might impede the development of such connectivity. When a human performs steady walking at a constant speed, a spinal central pattern generator (CPG) would be responsible for the rhythmic movements [65–67], so the connectivity between sensorimotor clusters would be diminished [36]. To avoid these low levels of cortical connectivity, transient gait training might be helpful, which could introduce deviations from steady-state for beneficial outcomes. Introducing the target speed to make walkers adjust their gait speeds and to force continuous body control on the user to minimize the error between the target and the real speeds could play a vital role in promoting connectivity in the cortical networks.

### Transient gaits

In addition to active walking at a target speed, which results in continuous body control, other walking tasks, such as accelerating and decelerating walking and stopping and starting walking, could also be implemented using the self-paced walking training system. In line with the goal of promoting cortical activity and connectivity, for example, such transient gaits could be performed by following a pet that is changing its speed or direction in a virtual reality (VR) system [68]. A variety of gait transients could be performed in a relatively small space and a safe environment at an affordable price because the self-paced training system consists of only the conventional linear treadmill, a display, and the Kinect. The described self-paced training system, therefore, could be utilized for gait rehabilitation with subjects who have difficulties in being trained outside or low accessibility to rehabilitation systems. In addition, other types of feedback systems can be implemented on the suggested self-paced treadmill walking system as required for gait training after stroke. Rather than the walking speed used in this study, gait asymmetry, for example, can also be provided as feedback to train these features of walking.

## Conclusion

This study focused on the changes in the levels of cortical activity from self-paced treadmill walking. The results should be confined to suggesting the potential of promoting neuroplasticity. Interestingly, not only the intact side but also the affected side showed a significantly higher level of cortical activation, mostly in the β and γ bands. In addition, to match the given target walking speed with the affected lower limb, the gait asymmetry was also decreased on the self-paced treadmill walking. Thus, it may be possible to promote regaining walking performance even when the affected lower limb is responsible for body control with the self-paced gait training system. To determine if neuroplasticity is promoted using the self-paced walking training system, a long-term clinical test needs to be conducted.

## Data Availability

Not applicable. But the datasets may be available from the corresponding authors on reasonable request.

## Abbreviations

BWSTT: Body weight-supported treadmill training
EEG: Electroencephalography
PMC: Premotor cortex
PFC: Prefrontal cortex
SMC: Supplementary motor cortex
SG: Supramarginal gyrus
SSC: Somatosensory cortex
AG: Angular gyrus
FAC: Functional ambulation categories
BBS: Berg balance scale
FES: Falls Efficacy Scale
10 mWT: 10-M walking test
GRF: Ground reaction force
HS: Heel-strike
TO: Toe-off
ASR: Artifact subspace reconstruction
EICA: Extended independent component analysis
IC: Independent components
AMICA: Adaptive mixture independent component analysis
ERSP: Event-related spectral perturbations
ERD: Event-related desynchronization
ERS: Event-related synchronization
ACA: Anterior cerebral artery
MCA: Middle cerebral artery
PCA: Posterior cerebral artery
